# Temporal constrained objects for modelling neuronal dynamics

**DOI:** 10.7717/peerj-cs.159

**Published:** 2018-07-23

**Authors:** Manjusha Nair, Jinesh Manchan Kannimoola, Bharat Jayaraman, Bipin Nair, Shyam Diwakar

**Affiliations:** 1 Amrita School of Biotechnology, Amrita Vishwa Vidyapeetham, Kollam, Kerala, India; 2 Department of Computer Science and Applications, Amritapuri Campus, Amrita Vishwa Vidyapeetham, Kollam, Kerala, India; 3 Center for Cybersecurity Systems and Networks, Amritapuri Campus, Amrita Vishwa Vidyapeetham, Kollam, Kerala, India; 4 Department of Computer Science & Engineering, State University of New York at Buffalo, Buffalo, NY, USA

**Keywords:** Temporal constrained objects, Constraint programming, Object-oriented languages, Declarative modelling, Neuron models

## Abstract

**Background:**

Several new programming languages and technologies have emerged in the past few decades in order to ease the task of modelling complex systems. Modelling the dynamics of complex systems requires various levels of abstractions and reductive measures in representing the underlying behaviour. This also often requires making a trade-off between how realistic a model should be in order to address the scientific questions of interest and the computational tractability of the model.

**Methods:**

In this paper, we propose a novel programming paradigm, called *temporal constrained objects,* which facilitates a principled approach to modelling complex dynamical systems. *Temporal constrained objects* are an extension of *constrained objects* with a focus on the analysis and prediction of the dynamic behaviour of a system. The structural aspects of a neuronal system are represented using objects, as in object-oriented languages, while the dynamic behaviour of neurons and synapses are modelled using declarative temporal constraints. Computation in this paradigm is a process of constraint satisfaction within a time-based simulation.

**Results:**

We identified the feasibility and practicality in automatically mapping different kinds of neuron and synapse models to the constraints of *temporal constrained objects*. Simple neuronal networks were modelled by composing circuit components, implicitly satisfying the internal constraints of each component and interface constraints of the composition. Simulations show that *temporal constrained objects* provide significant conciseness in the formulation of these models. The underlying computational engine employed here automatically finds the solutions to the problems stated, reducing the code for modelling and simulation control. All examples reported in this paper have been programmed and successfully tested using the prototype language called TCOB. The code along with the programming environment are available at http://github.com/compneuro/TCOB_Neuron.

**Discussion:**

*Temporal constrained objects* provide powerful capabilities for modelling the structural and dynamic aspects of neural systems. Capabilities of the constraint programming paradigm, such as declarative specification, the ability to express partial information and non-directionality, and capabilities of the object-oriented paradigm especially aggregation and inheritance, make this paradigm the right candidate for complex systems and computational modelling studies. With the advent of multi-core parallel computer architectures and techniques or parallel constraint-solving, the paradigm of *temporal constrained objects* lends itself to highly efficient execution which is necessary for modelling and simulation of large brain circuits.

## Introduction

Modelling complex systems using computer languages has spanned a wide range of domains: from organs and organ systems to weather and atmospheric turbulence to economic systems and social networks. While it is the responsibility of the programmer to choose an appropriate paradigm for the problem at hand, conventional languages are limited in their ability to provide the right framework for a broad range of problems. Models for complex problems tend to be large and unwieldy, and hence it is critically important that the programming language used to program such models not exacerbate the problem with inadequate support. In this regard, imperative languages require more effort on the programmer, in providing the detailed data representation and algorithms, needed to solve a problem. This adds another layer of software complexity, especially when the problem to be modelled is a highly complex one.

Declarative languages had their origins in 1960s and are useful in directly modelling a problem by stating the properties of solutions (Benhamou, Jussien & O’Sullivan, 2007). In constraint-based languages, programmers declaratively specify the relation between variables using constraints, and the task of solving/maintaining the constraints is the responsibility of the underlying constraint solvers (Freeman-Benson, Maloney & Borning, 1990). This approach provides the desired separation between the problem-specification phase and the problem-solving phase. In this paper, we present a compositional approach in constraint programming to model the structure and behaviour of complex biological systems using the concept of *temporal constrained objects* (Kannimoola et al., 2017).

*Temporal constrained objects* are an extension of the paradigm of *constrained objects* which has been studied for over three decades (Borning, 1981; Leler, 1987; Horn, 1993; Tambay, 2003) and provide a declarative approach to data abstraction using the concepts of classes, hierarchies and aggregation found in object-oriented languages (Lago & Artalejo, 2001; Reiner & Zimmer, 2017). Constrained objects also provide a declarative approach to behavioural specification using constraints within the class (Jayaraman & Tambay, 2002). Constrained objects have been used previously to model cellular behaviour (Covert, Famili & Palsson, 2003) and metabolic pathways in cells (Pushpendran, 2006) in the context of biological systems. Although constraint satisfaction problems were introduced originally as a static framework, the paradigm of *temporal constrained objects* allows a modeller to solve a broader class of problems. In *temporal constrained objects*, constraint-solving is integrated within a time-based simulation regime and is well-suited to problem domains that require ordinary differential equations or partial differential equations. Extensions to constraint programming frameworks, such as hybrid concurrent constraint programming (Gupta et al., 1995), have also proved to be useful in modelling constraint satisfaction problems with time-varying behaviours. *Temporal constrained objects* were successful in modelling highly dynamic systems such as vehicular networks (Kannimoola, Jayaraman & Achuthan, 2016) and firewalls (Kannimoola, Jayaraman & Achuthan, 2018). This paper applies similar modelling principles to neural microcircuits.

In this paper, we demonstrate how the paradigm of *temporal constrained objects* can be applied for modelling the structure and behaviour of a complex biological system. *Temporal constrained objects* are appropriate for systems whose behaviour is governed by physical laws. The adaptive and dynamic nature of neural circuits demands efficient modelling strategies to incorporate structural compositions of the constituents at various translational levels—from ion channels to neurons to networks and behavioural systems. This paradigm is suitable to model neural systems since it focuses on a component-based modelling approach, with individual components governed by invariant principles. For example, neurons’ and synapses’ signalling mechanisms and its non-linear dynamics are represented by membrane voltage models constrained by current and voltage laws, and are also known to be constrained by neuronal anatomy and interconnection between neurons (Gutkin, Pinto & Ermentrout, 2003). While building neural networks, the aggregation of different neuronal circuit elements was automatically addressed using internal and interface constraints, without imposing the relations explicitly from outside.

In this paper, the section ‘Background’ gives background about the programming language aspects of *temporal constrained objects* followed by the essential modelling principles of neural systems. Computational modelling of neural systems using *temporal constrained objects* is described in the section ‘Methods.’ We present a detailed case study of an implementation of neurons and the micro-circuitry of a rat cerebellum granular layer. The ‘Results’ section includes the results of modelling with *temporal constrained objects* as well as model validations and performance optimizations. The last two sections of the paper highlights the discussion followed by conclusions and remarks on future research directions.

## Background

### Programming methodology

Popular mainstream programming languages such as C, Java or Python require the programmer to specify detailed procedural instructions on how to solve a problem. In these languages, model specification and model implementation details are interwoven. In contrast, a declarative program specifies the expected result of a computation without explicitly detailing with the steps that must be performed to obtain that result. That is, declarative programming focuses more on what is to be computed, rather than how (Lloyd, 1994). In this paper we introduce a declarative programming paradigm called *temporal constrained objects* which integrates declarative constraints and constraint solving within the popular object-oriented paradigm for data abstraction.

A constraint is a declarative specification of a relation between variables and does not explicitly specify a procedure or algorithm to enforce the relation. In constraint programming, the programmer models the system as a set of variables over well-defined domains and states the problem as a set of constraints on these variables. The constraint solver enumerates the possible values of the variables and checks whether these enumeration leads to a solution or not, by a process called constraint satisfaction. Constraints have been used in the past for formulating many combinatorial problems, including search problems in artificial intelligence and operational research.

Object-oriented methods support component-based modelling where the whole system can be modelled incrementally using subsystems modelled previously. Although most mainstream languages that support object-oriented principles follow the imperative style of programming, object-oriented languages supporting declarative semantics have also been proposed (Lago & Artalejo, 2001). In *temporal constrained objects* the state of an object, i.e., the values of its attributes, is determined by a set of declarative constraints rather than by imperative procedures.

### Constraint-based and related systems

Among the first in the area of constrained objects was ThingLab (Borning, 1981), a constraint-based simulation laboratory designed for interactive graphical simulations. The Kaleidoscope ’91 language (Lopez, Freeman-benson & Borning, 1994; Govindarajan, Jayaraman & Mantha, 1996) integrated constraints and object-oriented programming for interactive graphical user interfaces. Kaleidoscope added constraint hierarchies, multi-methods and constraint constructors, as well as user-defined constraints, which were simplified by the compiler to primitive constraints that could be solved by a primitive solver. Bertrand (Leler, 1987) was another language aimed at graphics applications, which was extended by Bruce Horn in his constrained object language Siri (Horn, 1991). This language used the notion of event pattern to declaratively specify state changes: by declaring what constraints held after the execution of a method, and also specifying which attributes may and may not change during the method execution. This constraint imperative language uses constraints to simulate imperative constructs such as updating, assignment and object identity. Inspired by Kaleidoscope, the language Babelsberg (Felgentreff et al., 2015) was developed that integrates constraints with object-oriented systems. A Ruby extension has been developed wherein programmers could add constraints to existing Ruby programs in incremental steps. Another extension has been made accessible from the Smalltalk language to enable the dynamic management of constraints, and a similar approach was followed by integrating constraint programming into Java language environment (Hon & Chun, 1999). Being early approaches, they provide a limited capability for expressing constraints. Modelica (Fritzson, 2004) is a constrained object language for modelling and simulation in the engineering context. It supports numerical constraints in an object-oriented framework and uses the MATLAB engine for constraint solving. *Temporal constrained objects* presented in this paper also employs similar modelling principles.

### Existing neuroscience modelling platforms and tools

Modelling and simulation techniques are extensively used as powerful complements to hypothesis testing for experimental studies related to biological and physiological systems. Simulation engines, computing environments and languages help building the computational model of the system from mathematical models. Currently, a neuroscience modeller has wide choice of tools that support better integration and model reuse across multiple simulators. While simulators such as NEURON, GENESIS and NEST employ domain-specific custom scripting languages to isolate model specification from simulation code, interoperable scripting was supported in simulators like MOOSE and PyNN (Goddard & Hood, 1998; Hines & Carnevale, 1997; Diesmann & Gewaltig, 2001; Ray & Bhalla, 2008; Davison et al., 2009). NEURON uses the interpreted language hoc for simulation control and a high-level language Neuron model description language (NMODL) for describing models, where each line of NMODL is translated into equivalent C statements. Both GENESIS and NEST use high-level simulation languages to model neurons and synapses. While the simulation kernel of NEST is written in C++, Python commands are used to formulate and run neuron simulations with an extended code generation support using Cython (Zaytsev & Morrison, 2014). GENESIS also supports declarative modelling using a script-based language interpreter. These specialized tools are less verbose and can address different domain-specific modelling tasks in a computationally tractable way. All these simulators are software packages with several thousands of lines of code (LOC) and pose model exchange and interoperability problems. Although conceptual structure of modelling is commonly addressed in these simulators in a precise and concise manner, the simulation kernel of these tools uses object oriented and low level procedural code libraries.

Since conversion of models from one simulator to another is a non-trivial task, simulator-independent language initiatives facilitated model sharing and model reuse across multiple simulators. PyNN and MOOSE uses high level Python libraries and APIs to support simulator independent interface for neuron modelling. Apart from these attempts, XML based model specification languages have helped reduce the implementation and platform dependent biases in the modelling process. As a model representation language, systems biology markup language provides a common format for describing models and supports interoperability among different tools in computational biology (Hucka et al., 2003). NeuroML (Gleeson et al., 2010) uses distinctive XML schemas to represent the morphology (MorphML), channel conductance (ChannelML) and network properties (NetworkML) of neural systems. NineML (Raikov, 2010) uses XML based abstract object models and enable quick prototyping of neuron, synapse and network models using parameters for model variables, state update rules and mathematical descriptions. Low entropy model specification also follows a similar approach and are more flexible in defining and translating models (Cannon et al., 2014). Even though the XML-based model description frameworks reduce external software dependencies, they do not provide any details on how to simulate the models. XML schemas supports model exchange and automated validation of components but for better integration with existing simulators, these models should be easily translatable to multiple simulation platforms. The simulators need to either translate the models into equivalent internal models or use code generation through external or built in libraries. Spiking Neural Mark-up Language (SpineML), an extension of NineML uses simulator specific eXtensible Stylesheet Language Transformations (XSLT) templates to generate the simulation code (Richmond et al., 2014). The encoding of model details from XML format to the internal representation of the simulator completely depends on the data structure of the language selected for this translation. For example, mapping the NeuroML model description to the internal data structure of the simulators such as NEURON, GENESIS, PyNN or MOOSE is provided through simulator specific native scripts.

Although the aforementioned simulators hide the implementation complexity from the modeller either through GUI or through modules and scripting, the software complexity of the simulator increases while satisfying these requirements. The computational architecture of the simulators handled the complexity and provided interfaces to the end user. Since *temporal constrained objects* are founded on constraints, a model’s concept space (model specification) and computational space (simulation control) can be represented with less implementation complexity. For modelling multiple levels as in enzyme or biochemical kinematics to neurons and neural circuits, a constraint-based solver that could handle several models of differential equation style mathematical abstractions was attempted in this study.

Another motivation for our choice of the paradigm of *temporal constrained objects* is its amenability to parallelization. Modern programming paradigms are biased more towards many-core and multi-core programming. Current day simulation systems have become more reliant on high performance computing techniques. In computational neuroscience, a modelling study generally involves learning a domain specific language and then depending on the framework of this language to parallelize the programs. NEURON and GENESIS require modifications in the coding strategy to port the model to cluster computing environment while NEST almost transparently maps a model to multicore computers. Brian has little support for parallelization which limits its use for large scale systems (Goodman & Brette, 2008). The transition of a software to parallelization is easier with declarative paradigms (Darlington, Reeve & Wright, 1990). The advantage with parallel constraint programming is that no change is needed in the problem model for parallelization and the constraint framework handle the parallelization primitives. Parallel constraint programming frameworks exist (Rolf, 2011) which automatically parallelize the problem by dividing the task among several constraint solvers which perform parallel consistency and parallel search processes. Since consistency checking during constraint solving has similarities to single instruction multiple thread parallelism, GPU level parallelization of constraint propagation has been explored recently (Campeotto et al., 2014). Although beyond the limits of this paper, integrating parallelization with *temporal constrained objects* frameworks will benefit the neuroscience community to easily create abstract models which are automatically scalable.

### Temporal constrained objects

*Constrained objects* support object-oriented features, such as aggregation and inheritance, along with constraint-based specifications such as arithmetic equations and inequalities, quantified and conditional constraints. The programming language COB implements the constrained object paradigm (Jayaraman & Tambay, 2002). The COB language defines a set of classes, each of which contains a set of attributes, constraints, predicates and constructors. Every constrained object is an instance of some class whose outline is as follows.


*class_definition ::=* [ abstract] class *class id* [ extends *class id* ] {*body*}
       *body ::*=  [ attributes *attributes*]
             [ constraints *constraints*]
             [ predicates *predicates* ]
             [ constructors *constructors*]


A class may optionally extend another class and the attributes, constraints, predicates and constructors are all optional. Constraints specify the relation between attributes of the typed classes. *Constrained objects* support both primitive and user-defined attributes, and constraints may be simple, conditional, quantified, or aggregate (also see Supplementary Material for a complete specification of the grammar of the language) (Tambay, 2003).

*Temporal constrained objects* extend the basic paradigm of constrained objects to support time-varying properties of dynamic systems. Using *temporal constrained objects*, the structural aspects and the modularity of a system are modelled using encapsulation, inheritance and aggregation while the behavioural aspects are modelled through a rich set of constraints. The underlying computational engine performs logical inference and constraint satisfaction to enforce the behaviour automatically while each object is created.

One of the most useful features of *temporal constrained objects* for modelling temporal (or dynamic) behaviour is the *series variable*, declared as:

series *type variable_name* [*initialization*];



The series variable takes on an unbounded sequence of values over time, and temporal constraints are defined in terms of past and future values of the series variable. For every series variable v, the expression ‵v refers to the immediate previous value of v and v‵ to the next value. These operators can be juxtaposed (for example, ‶v and v‶) to refer to successive values of v in the past or future. A series variable may also be initialized by explicitly assigning values at specific time points.

**Figure 1:**
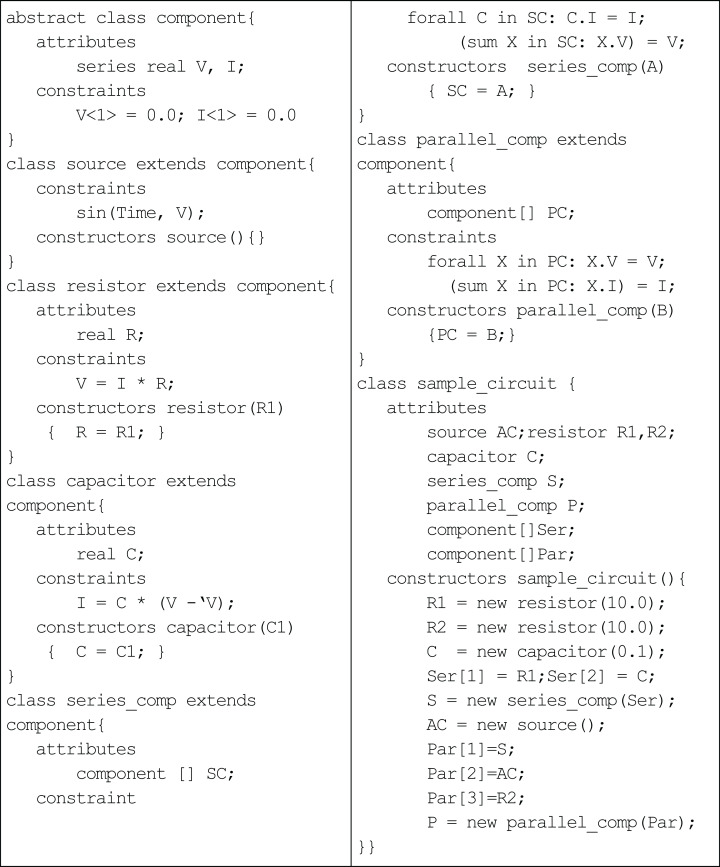
Temporal constrained object representation of AC circuit.

An example of an alternating-current (AC) circuit illustrates the basic constructs of the language (Fig. 1). The series attributes, voltage (*V*) and current (*I*), in the abstract component class holds the sequence of values at different instance of time. The source class generates the input voltage for the circuit, which varies sinusoidal with time. The classes resistor and capacitor inherit voltage and current from the component class. The constraints in each class define the laws of circuit elements: the resistor class incorporates Ohm’s law for resistance (Eq. (1)); and the capacitor class incorporates Ampere’s law for capacitance (Eq. (2)).

(1) $$V = IR$$

(2) $$I = C{{{\rm{d}}v} \over {{\rm{d}}t}}$$

The differential equation (Eq. (2)) is treated as difference equations in the capacitor class, i.e. the rate of change of voltage can be approximated by change in voltage in one unit of time. The parallel_comp and series_comp classes represent the two different ways of combining the components of the AC circuit. (Of course, non-series parallel circuits can also be defined, but the example in Fig. 1 pertains only to series-parallel circuits.) The constraints describe the resultant voltage and current for both kinds of circuits. For example, in class parallel_comp, the quantified constraint forall enforces that the voltage across every component in the parallel circuit is the same and equal to the voltage across the parallel circuit; and, the summation constraint sums up the currents through each component of the parallel circuit and equates it to the current through the *parallel* circuit. The class sample_circuit builds a series component with a resistor and capacitor and a parallel component consisting of the series component, a sinusoidal voltage source and a single resistor. In order to understand the execution of a TCOB program, TCOB provides a built-in variable Time, which represents the current time and is automatically incremented by one unit to record the progression of time. The default initial value for Time is equal to one unless a different value is specified by the user. The user may adopt any other discrete granularity for time by multiplying with a suitable scaling factor, e.g. MyTime = 0.01 * Time.

**Figure 2:**
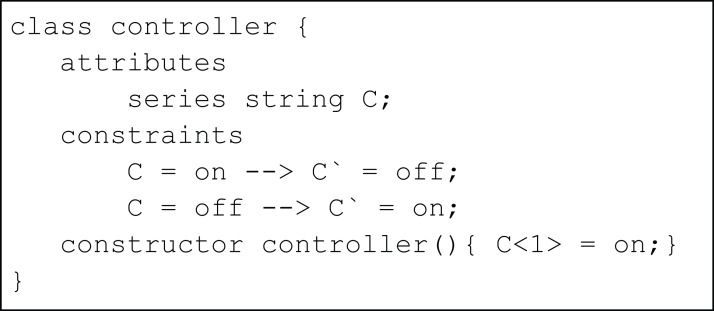
Simple controller using conditional constraints.

We use the example of an on-off controller (Fig. 2) to illustrate the basic concepts of conditional constraints in the TCOB. This is a simplified version of traffic light example from a previous paper (Kannimoola, Jayaraman & Achuthan (2016)). The variable C is declared using series keyword to model transitioning from on/off state in each time instance, specified by the conditional constraint symbol -->. When Time = 1, the value of C = on since this is the initial value of C as given in the constructor. At this time, the value of C‵, i.e. value of C at Time = 2 is set as off based on the first constraint in the program. In a similar manner, the second constraint determines values for C for off-on transition.

In this implementation, the TCOB programming environment consists of a compiler which translates the class definitions into equivalent predicates in SWI-Prolog which provides support for constraint-solving through its libraries. A more detailed description of the language is given in reference (Kannimoola et al., 2017).

### Electrical equivalent circuit of neurons

A single neuron is often conceptualised using the biophysics of a neuronal membrane, represented by resistance-capacitance (RC) low-pass filtering properties wherein the lipid bi-layer of the neurons is modelled as capacitance (*C*), the voltage-gated ion channels as electrical conductance (*g*) and the electrical gradient as reversal potentials. Then, basic voltage and current laws (Ohm’s and Kirchhoff’s laws) are employed to calculate the voltage across the membrane of the neuron. The cell membrane acts as a diffusion barrier and an insulator to the movement of ions. The lipid bi-layer of the membrane accumulates charges over its surface where the intracellular and extracellular solutions act as the conducting plates separated by the non-conducting membrane. The capacitive current *I_c_*, thus formed is

(3) $${I_c} = C{{{\rm{d}}V} \over {{\rm{d}}t}}$$

where *C* is the capacitance and *V* is voltage across the membrane. Ions flow into and out of the cell through ion channels. The flow of ions through these channels leads to resistive current flow into the neuron, which is represented using Ohm’s law:

(4) $${I_{{\rm{ion}}}} = V{G_{{\rm{ion}}}}$$

where *G*_ion_ is the conductance of ion across the membrane and *I*_ion_ is the ionic current.

The electromotive force acting over the ions as the battery of the circuit, when included, the ionic current can be represented as,

(5) $${I_{{\rm{ion}}}} = {G_{{\rm{ion}}}}(V - {E_{\rm ion}})$$

Total current flowing across the membrane is the sum of the capacitive and ionic currents:

(6) $${I_{{\rm{Total}}}} = {I_c} + {I_{{\rm{ion}}}}$$

At steady state, membrane voltage remains constant, which means that the net current into the neuron plus the net current out of the neuron must equal zero.

(7) $${I_{{\rm{Total}}}} = {I_c} + {I_{{\rm{ion}}}} = 0$$

When an input current *I*_Ext_ is injected into the cell, it further charges the capacitor and the current leaks through the membrane.

(8) $${I_{Ext}} = C{{{\rm{d}}V} \over {{\rm{d}}t}} + {I_{{\rm{ion}}}}$$

Larger number of open ion channels in the membrane decreases the resistance of the membrane due to increased ionic flow across the membrane. This results in an increase in conductance across the membrane. Ionic current flow through a neuron with Sodium, Potassium and Leak channels can thus be modelled as,

(9) $${I_{Ext}} = C{{{\rm{d}}V} \over {{\rm{d}}t}} + {G_{Na}}(V-{E_{Na}}) + {G_K}(V-{E_K}) + {G_L}(V-{E_L})$$

Passive electrical properties persist in the neuron if the current is a sub threshold current or a hyperpolarizing current. Neurons are known to communicate to each other using stereotyped electrical signals called action potentials or spikes. The generation of action potential in the neuron can be explained by modelling the activation of ion channels. Detailed conductance-based models like *Hodgkin–Huxley (HH) model* and simpler phenomenological models like *Izhikevich model* and *Adaptive exponential integrate and fire model* were used in this study to explain the spiking mechanisms of neurons.

## Methods

**Figure 3:**
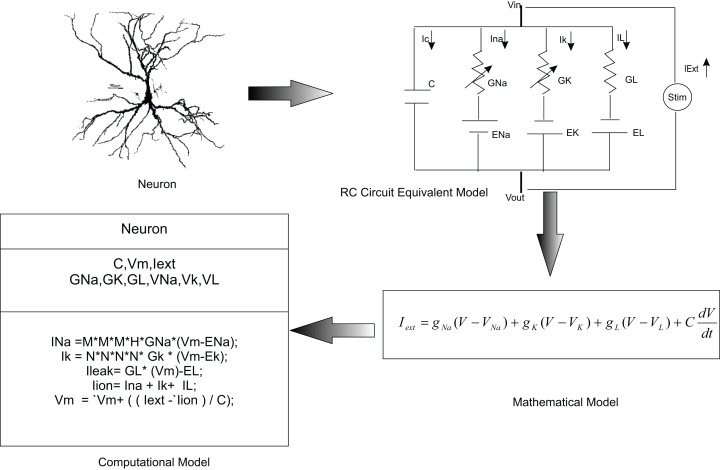
Computational modelling of neurons. It is a common practice to abstract a neuron as an RC circuit to describe the electrical properties of the neuron. Current flow in the neuron was mathematically modelled using this equivalent model. In the *temporal constrained object* approach, a neuron was represented as an abstract data type with attributes (state information) and constraints (rules of behaviour).

In this paper, we used *temporal constrained objects* to model the time-varying dynamics of a neural circuit as exhibited by the electrical activity of neurons and synapses. In modelling studies, elemental features of a neural system are abstracted into a conceptual model and are formalized into a mathematical form. A working embodiment of neural dynamics is created using computational models which is used to test and examine the modelled abstract. Chemical and electrical phenomena of neural systems were then simulated from mathematical representations (Fig. 3).

*Temporal constrained objects* allowed a direct implementation of the circuit model of a neuronal system. Initial focus was on modelling the component-based structure of the neural system by identifying the components, their functions and parameters. The main constituent of the neural system—the neuron—was modelled in two different formulations: (1) using voltage functions which are continuous over a time evolution (as in a HH model, described in ‘Modelling neurons using continuous time HH mechanisms’); and (2) using voltage reset functions which show piece wise state changes (as in AdEx and Izhikevich models, described in ‘Reconstructing neurons using voltage reset models’). ‘Modelling synaptic dynamics’ presents modelling the synaptic interactions between neurons using continuous functions of time.

In modelling neurons and synapses with *temporal constrained objects*, the biophysical mechanisms (abstracted as mathematical formulations in the models) were represented as constraints of the class. Continuous state changes of variables were represented using simple constraints while voltage reset functions were represented using conditional constraints. Once the fundamental components of a neural network were modelled, the interaction between these components in a network (‘Modelling neuronal interactions’ and ‘Modelling cerebellar microcircuits’) was incorporated. Biological fidelity of the models was tested to validate whether the model produces experimental outcomes.

### Modelling neurons using continuous time Hodgkin–Huxley mechanisms

Hodgkin–Huxley equations (Hodgkin & Huxley, 1952) model biophysical characteristics of a neuronal membrane in detail. This model exhibits various neuronal behaviour by the calculation of ionic current in their mathematical notation. A set of first-order differential equations specify the dynamics of membrane voltages and ion channels of the neurons. According to this model, the ionic current flow in Eq. (9) has been rephrased as,

(10a) $${I_{{\rm{Ext}}}} = C{{{\rm{d}}V} \over {{\rm{d}}t}} + {g_{{\rm{NaMax}}}}{m^3}h(V-{E_{\rm Na}}) + {g_{{\rm{KMax}}}}{n^4}(V-{E_{\rm K}}) + {G_L}(V-{E_L})$$

where the ionic conductance was modelled as voltage dependent quantity with the flow of ions regulated by physical gates which are either in their open state or in closed state. In (10a), *g*_NaMax_ and *g*_KMax_ are the maximum conductance of Na^+^ and K^+^ ions, *m* and *h* are the probabilities of the fast and slow subunits of *Na* channel being open and closed states and *n* is the probability of the subunit of a *K* channel being open. Dynamical properties of these gating variables are also represented using differential Eqs. (10b–10d).

(10b) $${{{\rm{d}}h} \over {{\rm{d}}t}} = {{\rm{\alpha }}_h}(1-h)-{{\rm{\beta }}_h}h$$

(10c) $${{{\rm{d}}n} \over {{\rm{d}}t}} = {{\rm{\alpha }}_n}(1-n)-{{\rm{\beta }}_n}n$$

(10d) $${{{\rm{d}}m} \over {{\rm{d}}t}} = {{\rm{\alpha }}_m}(1-m)-{{\rm{\beta }}_m}m,$$

Hodgkin–Huxley model represents a system of four differential Eqs. (10a to 10d) which are coupled via voltage *V* of the membrane. In the standard formulation of HH model, the rate constants α and β of the ionic gates are empirically determined as a function of membrane voltage *V* as

(10e) $${{{\rm{\alpha }}_m} = {{(2.5 - 0.1{\rm{}}V)} \over {{e^{2.5 \,- \,0.1{\rm{}}V}} - 1}},{{\rm{\beta }}_m} = 4{e^{ - {V \over {18}}}}}$$

(10f) $${{{\rm{\alpha }}_n} = {{(0.1 - 0.01{\rm{}}V)} \over {{e^{1 \,- \,0.1{\rm{}}V}} - 1}},{{\rm{\beta }}_n} = 0.125{e^{ - {V \over {80}}}}}$$

(10g) $${{\rm{\alpha }}_h} = 0.07{\rm{ }}{e^{-{V \over {20}}}},{{\rm{\beta }}_h} = {1 \over {{e^{3\,-\,0.1{\rm{ }}V}} + 1}}$$

**Figure 4:**
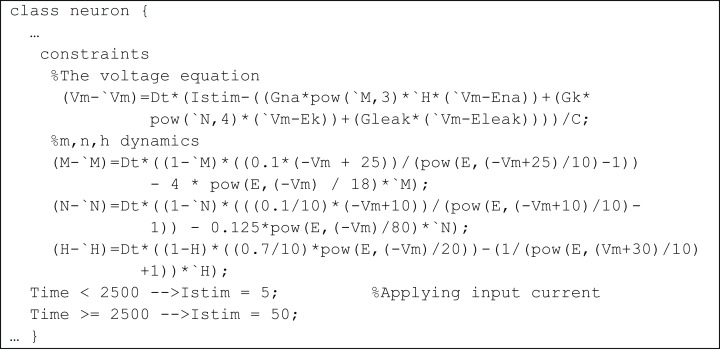
Representation of Hodgkin–Huxley model as a TCOB class.

In *temporal constrained object* implementation of the HH model, the properties of neurons, such as maximum channel conductance, reversal potential, or capacitance of the cell were represented as attributes of the class. Dynamically changing membrane voltage, the gating variables and the input current values were represented as *series* variables. The mathematical equations representing the relation between these variables were represented using quantified constraints and their initial values were represented using simple constraints (Fig. 4). Objects of the class can be created using creational constraints.

The membrane potential in the HH model varies as a continuous function over time and the numerical integration require that the values should be specified over a time evolution. In traditional object-oriented languages, the behaviour of the model can be enforced using member functions in the class which are to be explicitly called during execution. In the *temporal constrained object* based formulation, the differential equations are converted into difference equations and the simulation evaluated the constraint at every time step. This facilitates a modular representation, where the model behaviour is implicitly enforced during constraint satisfaction.

### Reconstructing neurons using voltage reset models

Detailed conductance-based models (as in ‘Modelling neurons using continuous time Hodgkin–Huxley mechanisms’) explain the behaviour of the neurons at a mechanistic level. Since such models have higher computational costs for complex circuits, we adopted two mathematical abstractions: Izhikevich model and Adaptive Exponential Integrate and Fire model (Izhikevich, 2003; Brette & Gerstner, 2005).

The Izhikevich model is expressed as:

(11a) $${{{\rm{d}}V} \over {{\rm{d}}t}} = 0.04{\rm{}}{V^2} + {\rm{}}5v + 140 - u + I$$

(11b) $${{{\rm{d}}u} \over {{\rm{d}}t}} = a\left( {bV - u} \right)$$

If

(11c) $$u > 30mV,{\rm{}}V = C{\rm{}},{\rm{}}u = u + d$$

In the two-dimensional set of ordinary differential equations of the Izhikevich model (Eqs. (11a)–(11c)), *V* is the membrane potential, *u* is the recovery variable, *a* represents the time scale of this recovery variable, *b* represents the sensitivity of *u* towards sub-threshold fluctuations in *V*, *C* represents the after spike reset value of *V*, *d* represents the after spike reset value of *u*. This implementation of a canonical model includes a nonlinear term *V^2^*, and has been shown to reproduce multiple behaviours of neurons such as spiking, busting, adaptation, resonance and mixed mode behaviours.

The Adaptive-Exponential Integrate and Fire (AdEx) model is expressed as:

(12a) $${{{\rm{d}}V} \over {{\rm{d}}t}} = {{{g_L}\,{\rm{*}}\,\left({V-{E_L}} \right) + {g_L}\,{\rm{*\Delta }}\,T{\rm\,{*}}\,\exp \left({{{V-{V_t}} \over {{\rm{\Delta }}T}}} \right)-{I_{{\rm{syn}}}}-w} \over C}$$

(12b) $${\tau _w}{{{\rm{d}}w} \over {{\rm{d}}t}} = a*\left( {V - {E_L}} \right) - w$$

If

(12c) $$V > 0mV,{\rm{}}V = {V_r},{\rm{}}w = w + b$$

This model (Eqs. (12a)–(12c)) represents spiking dynamics (Eq. (12a)) and the adaptation in the firing rate of the neuron (Eq. (12b)) with *V* representing the membrane voltage, *C* is the membrane capacitance, *g_L_* is the leak conductance, *E_L_* is the resting potential, Δ*T* is the slope factor, *V_t_* is the threshold potential, *V_r_* is the reset potential, *I*_syn_ is the synaptic current, τ*_w_* is the adaptation time constant, *a* is the level of sub-threshold adaptation and *b* represents spike triggered adaptation. The model implementation follows the dynamics of a RC circuit until *V* reaches *V_t_*. The neuron is presumed to fire on crossing this threshold voltage and the downswing of the action potential is replaced by a reset of membrane potential *V* to a lower value, *V_r_*.

**Figure 5:**
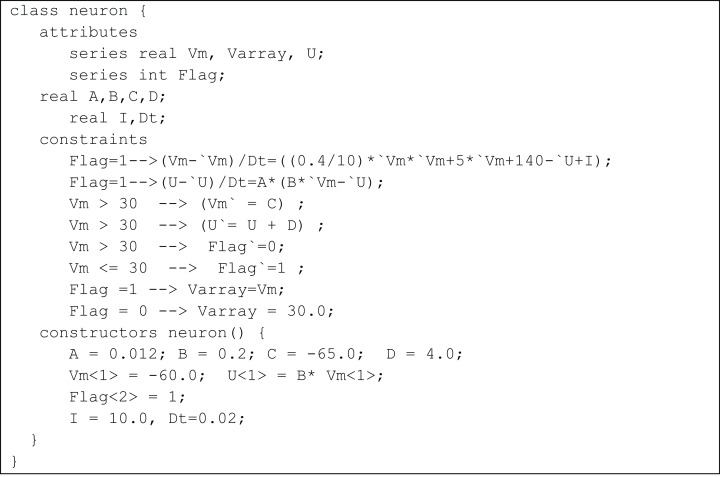
Izhikevich model represented as a TCOB class.

Since AdEx and Izhikevich models do not contain continuous-time equations, the membrane dynamics and its reset mechanisms in TCOB models were represented using conditional constraints. For example, Fig. 5 shows the class definition for an Izhikevich model. In the model, the membrane voltage does not change as a continuous function of time. The auxiliary reset of the membrane voltage *v* and the membrane recovery variable *u*, required a discontinuous change during constraint solving, the value being independent from the value of the variable in the previous time step. This change was controlled using the value of a series variable ‘*flag*,’ and using conditional constraints. It should be noted that this is not an *imperative* update of a state variable but rather the *declarative* specification of a value at a new point in time. Since the membrane potential *Vm* diverges towards infinity at a particular step and *Vm* need to be reset before this step, another series variable *Varray* was used to store a finite maximum upswing value for *Vm.*

AdEx type neurons were also modelled similarly (See Supplementary Material for the complete class definition).

### Modelling synaptic dynamics

The neurotransmitter release and intake mechanisms of the pre- and post-synaptic neurons were modelled using synaptic conductance *g*_syn(*t*)_. The changes were represented as continuous-time equations which represented the fluctuations in synaptic conductance changes attributed to current flow (Destexhe, Mainen & Sejnowski, 1998). The synaptic currents were calculated as the difference between reversal potential and membrane potential (Eq. (13)).

(13) $${I_{{\rm{syn}}\left(t \right)}} = {g_{{\rm{syn}}\left(t \right)}}\left({V-{E_{{\rm{syn}}}}} \right)$$

where *g*_syn(*t*)_ = *g*_max_ · *g*_(*t*)_, and *g*_max_ is the maximal conductance of the synapse, *g*_(*t*)_ is the time course of synaptic channel conductance, *I*_syn(*t*)_ is the synaptic current, *E*_syn_ is the synaptic reversal potential and *V* is the membrane voltage. The time course of channel conductance *g*_(*t*)_ was modelled using different mathematical formulations (Roth & van Rossum, 2009). For example, reconstructing exponential synapses biophysically included an instantaneous rise of *g*_syn(*t*)_ from 0 to *g*_max_ at time *t*_0_ (the time at which spike occurs) followed by an exponential term specifying the decay, with a time constant τ (Eq. (14)).

(14) $${g_{{\rm{syn}}\left( t \right)}} = {g_{{\rm{max}}}}.{e^{ - \left( {{{t - t0} \over {\rm{\tau }}}} \right)}}$$

The exponential synapse model approximates synaptic dynamics with the rising phase shorter than the decay phase. A single-exponential function is a reliable approximation for several synapses where the channels instantaneously transit from closed to open states. Alpha synaptic models have been used for synapses with finite duration rising phases (Eq. (15)) while in double-exponential synapse model, both rise time and decay time of the synapses were modelled separately (Eqs. (16a)–(16c)).

(15) $${g_{{\rm{syn}}\left( t \right)}} = {g_{{\rm{max}}}}.{{t - t0} \over {\rm{\tau }}}.{e^{{{1 - \left( {t - t0} \right)} \over {\rm{\tau }}}}}$$

(16a) $${g_{{\rm{syn}}\left( t \right)}} = {g_{{\rm{max}}}}\,.\,{f_{{\rm{norm}}}}{\rm{}}\,.\left( {{e^{ - \left( {{{t - t0} \over {{{\rm{\tau }}_{{\rm{decay}}}}}}} \right)}} - {e^{ - \left( {{{t - t0} \over {{{\rm{\tau }}_{{\rm{rise}}}}}}} \right)}}} \right)$$

(16b) $${f_{{\rm{norm}}}} = {1 \over {\left( {{e^{ - \left( {{{{{\rm{t}}_{{\rm{peak}}}} - t0} \over {{{\rm{\tau }}_{{\rm{rise}}}}}}} \right)}} + {e^{ - \left( {{{{{\rm{t}}_{{\rm{peak}}}} - t0} \over {{{\rm{\tau }}_{{\rm{decay}}}}}}} \right)}}} \right)}}.$$

(16c) $${{\rm{t}}_{{\rm{peak}}}} = {t_0} + {{{{\rm{\tau }}_{{\rm{decay}}}}\,.\,{{\rm{\tau }}_{{\rm{rise}}}}} \over {{{\rm{\tau }}_{{\rm{decay}}}} - {{\rm{\tau }}_{{\rm{rise}}}}}}\,.\,\ln {{{{\rm{\tau }}_{{\rm{decay}}}}} \over {{{\rm{\tau }}_{{\rm{rise}}}}}}$$

In *temporal constrained object* based implementation, synapses were also modelled using classes. Continuous conductance changes of the synapses were represented as constraints in each constituent class. Conditional constraints were used to represent the conductance changes before and after the stimulus onset. Synaptic currents were calculated by automatically evaluating the constraints in each class.

### Modelling neuronal interactions

Behaviour of sub-cellular or cellular components in biological neural circuits is not entirely sufficient to understand network function, since the dynamics and complexity of the neural systems are known to be nonlinear (Koch & Segev, 1998) (also see Supplementary Material, Section 4). In the bottom-up modelling of brain circuits, challenges remain in assessing how the properties of individual neurons combine together to produce the emergent behaviour at the circuit and translational levels. In designing large circuit models, new rules of interaction may emerge from underlying principles. Here, TCOB like frameworks offer a natural way to express the interactions by identifying and implementing the constraints.

**Figure 6:**
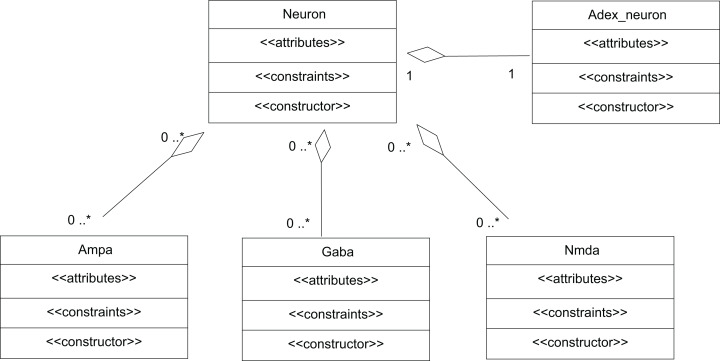
UML representation of TCOB implementation of a Neuron with synapses. A single neuron is represented as an aggregate of a neuron model and model synapses.

A network of neurons was modelled in TCOB, where each neuron in the network was simulated with different number of excitatory and inhibitory synaptic inputs. Excitatory synapses were modelled using AMPA and NMDA synaptic dynamics where inhibitory synapses were modelled using GABA synaptic dynamics (McCormick, Wang & Huguenard, 1993). All neurons were modelled as complex objects, i.e. consisting of other constrained objects with its own internal attributes and constraints (Fig. 6).

**Figure 7:**
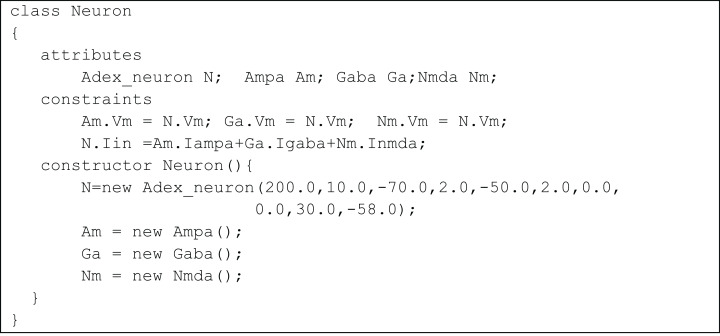
TCOB representation of Neuron with Synapse.

The class Neuron creates instances of the classes Adex_neuron to include the neuron model and the classes Ampa, Gaba and Nmda to model the synapses. Fig. 7 represents the TCOB model for UML class diagram in Fig. 6.

The modelled neuron receives synaptic inputs through one *AMPA*, one *NMDA* and one *GABA* synapse. The total input current (*I_in_*) to a neuron was set as the sum of its synaptic currents using the constraint:

N.Iin =Am.Iampa+Ga.Igaba+Nm.Inmda;



This constraint automatically enforces the relation between change in membrane voltage of the neuron and the synaptic inputs it receives. A cluster of such neurons were simulated by creating an array of TCOB objects. Constraint solving and evaluation of these objects utilized the implicit parallelization of constraints from the constraint store (Hutchison & Mitchell, 1973).

### Modelling cerebellar microcircuits

As a test of viability to use *temporal constrained object* based models for neural modelling, firing patterns of several types of known neurons of the cerebellum were reconstructed as in a previous study (Nair et al., 2014). Single neurons of Wistar rat cerebellum were modelled and attempted mathematical reconstruction of small scale cerebellar microcircuits. Associated with motor tasks and motor adaptation (Kandel et al., 2000), cerebellum receives inputs from major motor regions of the brain and gives feedback to these sources. The significantly large number of granule cells in the input layer of the cerebellum distinguishes cerebellum from the rest of the nervous system. The computational significance of these neurons has been a topic of interest and granule cell has received recent attention in computational modelling studies attributed to the numerosity, its electronically compact structure, simpler dendritic arbor and the signal recoding computations that it performs on the inputs that it receive from different brain regions (Diwakar et al., 2009; Solinas, Nieus & D’Angelo, 2010; D’Angelo, 2011). To reconstruct all the computational elements of the cerebellar circuitry and their convergence-divergence ratios, computationally effective methods may be needed to model circuit functions (Markram, 2006).

**Figure 8:**
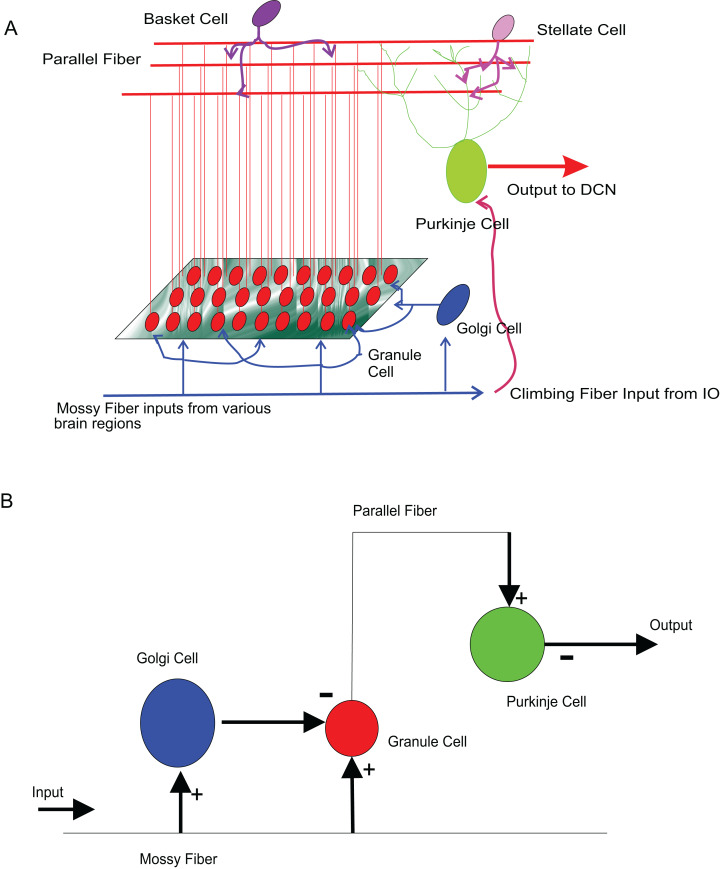
Modelling sample cerebellar microcircuits. (A) Circuits in the cerebellum: cartoon representation of interconnections in the input-output pathway of the cerebellum. (B) Cellular components of the microcircuit reconstructed: Granule neuron, Golgi neuron and Purkinje neuron, receiving inputs through excitatory (+) and inhibitory (−) synapses.

**Figure 9:**
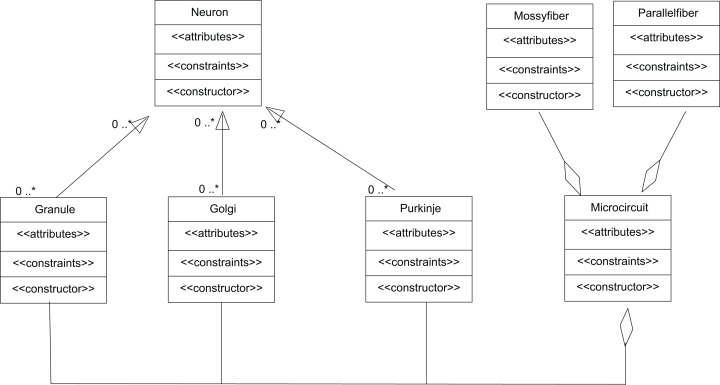
UML representation of *temporal constrained object* implementation of the microcircuit. *Granule*, *Golgi* and *Purkinje* classes inherit from the class *Neuron*. The classes *Mossy Fiber* and *Parallel Fiber* represented inputs to the neurons. In the implementation, the microcircuit consisted of an aggregation of the neurons and synapses.

The input to cerebellum is through mossy fibres innervating the granule and Golgi neurons (Fig. 8A). While Golgi neurons inhibit granule neurons via a feed-forward inhibition, the axons of granule cells extend as parallel fibres and excite Purkinje neurons. As in experiments (Diwakar et al., 2009), modelled granule neuron receives on an average four excitatory and four inhibitory connections. In this paper, a small scale granular layer circuitry with Purkinje neurons was modelled with *temporal constrained objects* (Fig. 8B) using classes granule, golgi, purkinje, mossyfiber and parallelfiber respectively. A model neuron inherited from the Neuron class and was represented using AdEx dynamics. Excitatory synapses were modelled using *AMPA* kinetics and inhibitory synapses were modelled using *GABA* kinetics. In the implementation, the microcircuit consisted of an aggregation of the neurons and synapses (Fig. 9).

**Figure 10:**
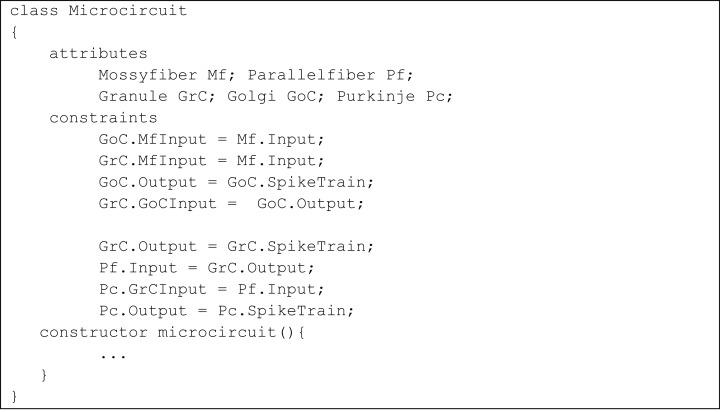
Representation of cerebellar microcircuit.

The *temporal constrained object* model of the microcircuit allowed computing the spike-train responses of constituent neurons. The class Microcircuit (Fig. 10) modelled the rat granular layer neural circuit (also see UML flow diagram in Fig. 9). In the model, granule and Golgi neurons received mossy fibre inputs and the change in membrane potential of Golgi and Purkinje neurons were automatically computed by satisfying internal and interface constraints.

Initial inputs to granule and Golgi neurons were provided by mossy fibre. This was represented in the constraints:

GoC.MfInput = Mf.Input;
GrC.MfInput = Mf.Input;



The variable SpikeTrain holds the response train of each neuron, generated as a result of model evaluation.

The Golgi neuron output is applied to granule neuron using the constraints

GoC.Output = GoC.SpikeTrain;
GrC.GoCInput = GoC.Output

such that the granule neuron receives both excitatory input through mossy fibres and inhibitory input through Golgi neurons.

In the next level of processing, output from the granular layer is applied as the parallel fibre input into Purkinje neurons using the constraints:

GrC.Output = GrC.SpikeTrain;
Pf.Input = GrC.Output;
Pc.GrCInput = Pf.Input;

After evaluating the constraints of Purkinje neuron, the entire output from the microcircuit was made available as:

Pc.Output = Pc.SpikeTrain;

The constraints highlighted above can be viewed as the interface constraints of the models while each object in the microcircuit class has its own internal constraint to be satisfied while object creation. The model evaluations were performed automatically while the constructor of the microcircuit class is called.

The entire code with the programming environment is available freely at https://github.com/compneuro/TCOB_Neuron.

## Results

### Temporal spike train firing patterns in neurons

**Figure 11:**
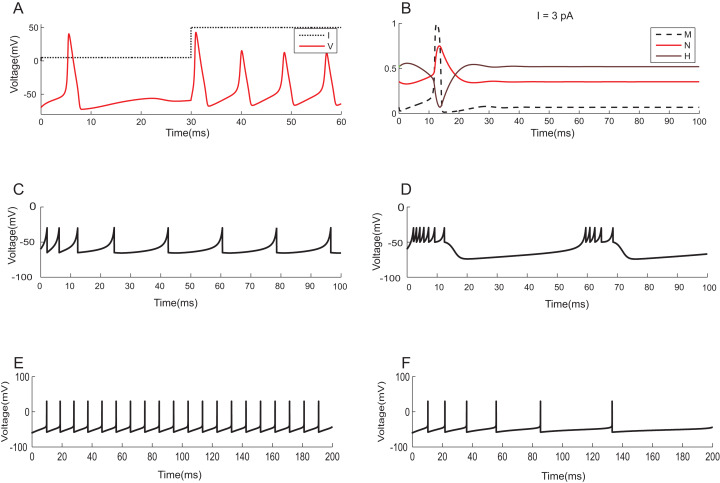
Modelling the spiking mechanisms of neurons. Simulation times are in millisecond scale matching slice recordings (Hodgkin & Huxley, 1952). The initial resting membrane potential was kept at −70 mV. (A) Firing of HH neuron for current stimuli of various amplitudes. The implementation showed repetitive firing behaviour for input current of six pA onwards. Firing rate of the neuron depended on the intensity of the injected current. Firing rate increased as the depolarizing current increases. (B) Channel Gating parameters *m, n, h* of the model for input current of three pA. *m* changed faster than *h* or *n*. During hyperpolarization *m, h* and *n* returned towards resting values. (C) Regular spiking behaviour shown by most typical neurons in the cortex reproduced using Izhikevich model. (D) Chattering: Stereotypical bursts of closely spaced spikes reproduced using Izhikevich model. (E) Tonic spiking with sharp reset showed the behaviour of certain constantly active neurons, modelled using AdEx equations. (F) Adaptation behaviour of certain neurons showing the reduction in firing frequency, modelled using AdEx equations.

To demonstrate the effectiveness of constraint evaluation results against state-of-the-art simulations, the output of TCOB models were recorded using TCOB predicates. Using standard values for model parameters, voltage plots of continuous-time HH models and voltage-reset models, Izhikevich and AdEx, were reproduced (Naud et al., 2008). In HH models, computationally reconstructed action potential peaked at a voltage of +40 *mV*, followed by hyper-polarization following which the resting potential was re-established (Hodgkin & Huxley, 1952) (Fig. 11A). As in experiments, when the injected current in the model was insufficient to depolarize the membrane, no action potential was generated. In our implementation, a minimum threshold non-zero current was observed for which the HH model demonstrated repeated firing, and the firing frequency increased with the increase in intensity of the input. The plot of HH gating variables depicted the behaviour of channel activation and inactivation (Fig. 11B). Izhikevich and AdEx models were also stimulated with input currents to reproduce various firing behaviour of different neuron types in the brain (Figs. 11C–11F).

**Figure 12:**
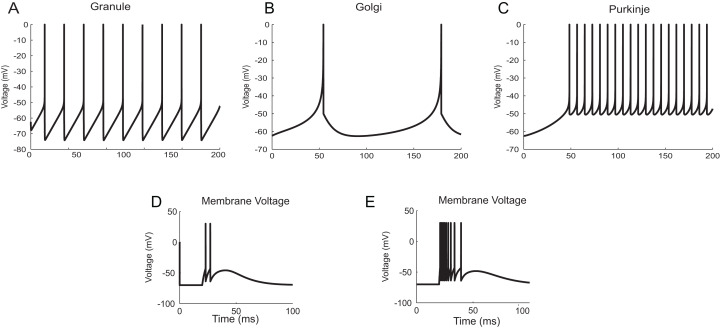
Modelling cerebellar neurons. (A–C) Granule, Golgi and Purkinje neuron responses for current clamp protocol (*I* = 10 pA), modelled using AdEx equations. (D and E) Response of granule neurons for in vitro like (left) and in vivo like (right) spike inputs.

The neuron and synapse models implemented using *temporal constrained objects* were parameter optimized to reproduce the firing behaviour of different neuron types present in the cerebellum (Medini et al., 2014; Nair et al., 2014) under current clamp experiments (D’Angelo et al., 1995; Bezzi et al., 2004), during in vitro behaviour (as seen in brain slices) and during in vivo behaviour (as seen in anaesthetized rats) for inputs through mossy fibres (Diwakar et al., 2009). Single spike inputs were applied through the synapses to model in vitro inputs while small burst inputs (e.g., five spikes per burst) was used to model in vivo inputs (Roggeri et al., 2008). The modelled responses of granule, Golgi and Purkinje neurons using AdEx neuron models for 10 pA input current are shown in Figs. 12A–12C (Medini et al., 2014). The modelled responses of granule neurons during in vitro inputs and in vivo inputs are shown in Figs. 12D and 12E.

### Synaptic dynamics resolved using TCOB

**Figure 13:**
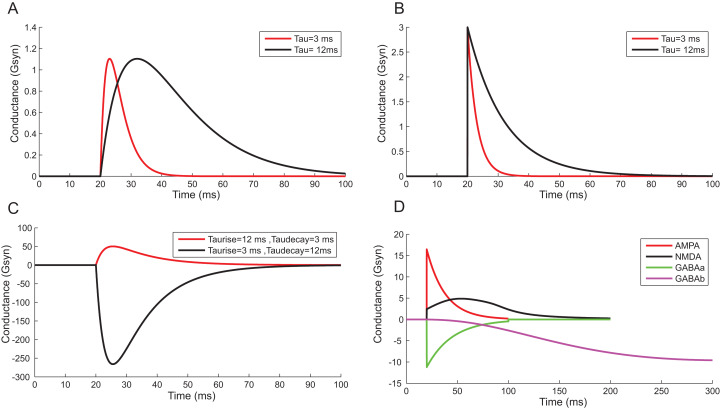
Reconstructing Synaptic Dynamics using *temporal constrained objects*. (A) The conductance changes in alpha function for τ = 3 ms and τ = 12 ms. (B) Synaptic conductance modelled using single exponential function for τ = 3 ms and τ = 12 ms. (C) Synaptic conductance modelled using double exponential function. (D) Modelled conductance changes for AMPA, NMDA, GABA_A_ and GABA_B_ synapses for input spike at time *t* = 20 ms.

Using a conductance-based synaptic mechanism, neurons were excited synaptically using pre-synaptic spike trains. Alpha function reproduced the post-synaptic conductance of synapses with a finite rise time (Fig. 13A). Instantaneous rise and exponential decay of membrane potential were modelled using single exponential synapses (Fig. 13B). A closer approximation of post-synaptic dynamics was obtained by employing double exponential models. Fluctuations of synaptic conductance were approximated using rise time and decay time of conductance change independently (Fig. 13C). The activation kinetics of excitatory and inhibitory synaptic receptors was modelled using AMPA, NMDA, GABA_A_ and GABA_B_ receptor behaviour (Fig. 13D). In the models, AMPA channels mediated the fast-excitatory transmission and were characterized by fast rise time and decay time for the conductance values. Significantly slower NMDA channel modelled related to modifications of synaptic efficacy and temporal summation of currents. In the implementations, the two primary inhibitory receptor kinetics; GABA_A_ and GABA_B_ modelled the fast and slow time course of inhibitory interactions.

### Correctness of computations and performance improvements in TCOB models

**Figure 14:**
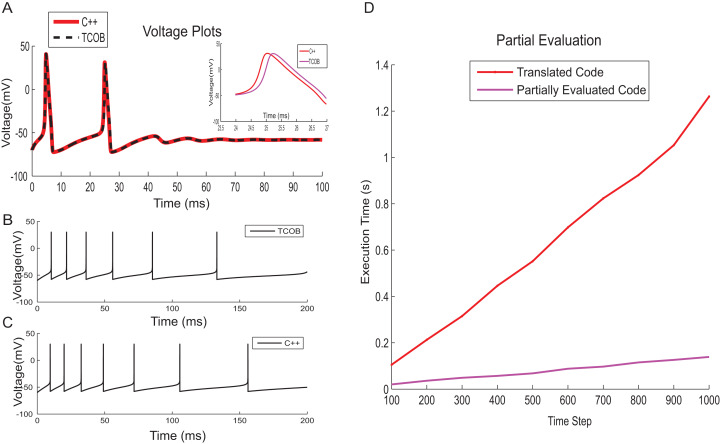
Correctness of computations and performance improvement. (A) HH model voltage traces simulated using C++ and TCOB for 6 pA input current. Inset shows the area of voltage plot in which absolute error was high. (B) Firing patterns for spike train adaptation of neurons modelled with AdEx model and implemented in TCOB. (C) Firing patterns for spike train adaptation of neurons modelled with AdEx model and implemented in C++. (D) Time step vs. execution time plot for HH model to show the improvement in performance time for partially evaluated code.

To test numerical accuracy of TCOB for floating point computations, the results of numerical integration of TCOB were compared with imperative C++ implementation. Fourth-order Runge–Kutta integration technique was employed for solving differential equations in the imperative implementation while numerical approximation using Euler approach was used for the current TCOB models. It has been observed that the membrane voltage traces produced by both approaches were approximately similar for the entire time evolution (Figs. 14A–14C). In this paper, we present the core concepts of TCOB and we have not discussed library support for the language. It is straightforward to make standard solvers for differential equations, such as Runge–Kutta, as library functions so that the programmer does not have to define them. However, these solvers need to be ‘programmed’ by the end user, the specification occurs at a much higher level than would be the case in an imperative language such as C or C++.

Although LOC are not always a definitive measure of software complexity, the declarative approach of a *temporal constrained object* model significantly reduced coding time, making the model more compact and closer to the problem specification, and hence also easier to understand, similar to scripting languages used in computational modelling. In comparison with the C++ version of the HH model, which required about 300 LOC, the *temporal constrained object* version was implemented in just 30 LOC.

TCOB compiler translates the *temporal constrained objects* program into Prolog code which can be executed directly under SWI-Prolog with a CLP(R) library. The potential sources of performance inefficiency of TCOB are due to the layers of predicate calls arising from the systematic translation and also due to the repeated checking of consistency in the underlying constraint store. To alleviate these inefficiencies, we adapted and extended a partial evaluation technique (Kannimoola, Jayaraman & Achuthan, 2016) for optimized code generation of constrained object programs that was first presented in Tambay (2003). Given the translated Prolog program of TCOB and a top-level goal, the partial evaluator outputs the list of constraints that need to be solved for the given goal. The partial evaluation process makes our implementation independent from constraint solver. On an Intel i7 processor-based desktop computer with 16 GB memory, the partially evaluated code for HH model executed approximately six times faster than the corresponding normal code (Fig. 14D).

## Discussion

*Temporal constrained objects* were able to successfully recreate substantive classes of neuroscience models related to time-varying behaviour using a declarative approach. With large-scale brain circuit models in mind, the compositional modelling requirement was tested by mathematically reconstructing the constituent components of neuronal network, i.e., neurons and synapses. Models that followed continuous-time functions, such as the HH type neuron model, as well as different synaptic models were implemented in *temporal constrained objects* easily. Special mention must be made of how *temporal constrained objects* were able to express voltage-reset models which exhibit piecewise continuous-time behaviour through a constant reset of the voltage. These models exhibit both discrete and continuous state changes, in contrast to the continuous-time behaviour of HH type neuron models. Such systems that exhibited discontinuous behaviour or choice, such as the Izhikevich- and AdEx-type neuron models, required injecting a discontinuous change during constraint-solving. *Temporal constrained objects* were able to elegantly support this capability using series variables and conditional constraints, i.e., whenever the condition was met for resetting the voltage, the value of the series variable at the applicable point in time is set appropriately. It should be noted that this is not an imperative update of a state variable but rather the declarative specification of a value at a new point in time of the series variable. Both continuous and voltage reset behaviour of the models implemented in *temporal constrained objects*, generated typical firing patterns of different neurons. Even though we have used manually defined synaptic connections in the example scripts, conditional constraints and dynamic typing in TCOB enables to use dynamic construction of objects based on the various conditions of the networks. Since *temporal constrained objects* also supports unbounded arrays, neurons can be programmed to receive dynamic stimuli generated from constraints. Since TCOB programming environment use SWI-Prolog built-in predicates to create random numbers, synaptic jitter and other forms of randomness in the network can also be modelled easily. Having been able to test that the tractability of employing *temporal constrained objects* as arbitrary neuron models, we perceive that *temporal constrained object* implementations allow continuous functions as in HH models or discrete state change of functions as in voltage reset models.

Constraint-based systems accumulate and aggregate the partial information from the set of constraints. Moreover, the order of constraint specification does not depend on the final result, which permits the incremental addition of information to the constraint store at any point of execution without worrying about the computation state. *Temporal constrained object*-like frameworks are well-suited for modelling systems from which useful inferences are to be derived based on partial or incomplete information available at the current stage. In computational neuroscience of neurons and circuits, this feature enables the modeller to process information incrementally at each functional zone with in specialized brain circuits.

## Conclusion

In our *temporal constrained object* implementation, the modelled neural responses reproduced biologically validated and relevant outputs as observed in experiments. The identification of global constraints of the system are to be tested on a larger scale, i.e. at the population level. Debugging network models in such frameworks has been changed to a constraint satisfaction problem where the constraint-solving algorithms operate on the constraint representation of the network variables, their domain and a set of relations between these variables. Successively imposing constraints with the level of details expected makes the system automatically scalable in terms of its software representation.

Although the current implementation of *temporal constrained objects* is not most efficient to compare with specialised simulators available for computational neuroscience, it is hoped that with novel re-implementation of the *temporal constrained objects* programming platform, we would be able to express models for large-scale reconstructions in the future. With new computing architecture and multiple core GPUs and CPUs, it is crucial to consider declarative modelling strategies that allow implicit parallelization. This re-implementation, however, pilots a general-purpose constrained object-based programming paradigm to declaratively express both the concept space and computational space of neuron models where the model evaluations are automatically handled by the computational engine.

While declarative languages provide a much higher level of abstraction and are more scalable, the execution of declarative programs under early implementations faced performance bottlenecks. Since slight changes in the constraint formulation may lead to unpredictable changes in the performance of the system, stability of constraint formulation of a model has always been challenged. Similar limitations exist while applying cost optimization techniques (Barták, 1999). Over the years, with advances in compiler and hardware technologies, the performance of declarative programs improved significantly, but is still not equal to that of imperative programs such as C or C++. Detailed performance measures were introduced to reduce the execution time by using methodologies such as compile-time optimization and partial evaluation. With these improvements, we feel that our proposed paradigm of *temporal constrained objects* is a good candidate for modelling small- to medium-scale brain circuit structures. In order to build constrained-object-based simulators for large-scale networks, we propose studying the parallelization of *temporal constrained objects. Temporal constrained objects* with parallelization are a promising approach for representing the emergent properties of systems that otherwise is too complex to model at multiple translational levels.

## Supplemental Information

10.7717/peerj-cs.159/supp-1 Supplemental Information 1 Supplementary text and code for temporal constrained objects for modelling neuronal dynamics. Code samples and descriptions of modeling implementation of neurons and synapses as temporal constrained objects.

## Additional Information and Declarations

### Data Availability

The following information was supplied regarding data availability: GitHub: https://github.com/compneuro/TCOB_Neuron.
