# Supplementary material for: Temporal constrained objects for modelling neuronal dynamics
Source: PeerJ Comput Sci. 2018 Jul 23;4:e159. doi: 10.7717/peerj-cs.159 (PMC7924700; doi:10.7717/peerj-cs.159)
Supplement: Supplemental Information 1 — Code samples and descriptions of modeling implementation of neurons and synapses as temporal constrained objects. [file peerj-cs-04-159-s001.docx]

**Supplementary material**

1. **TCOB Grammar**

class_definition ::= [ **abstract** ] class *class_id* [ **extends** *class_id* ]{ body }

body  ::= [ **attributes** attributes ]

          [ **constraints** constraints ]

          [ **predicates** predicates ]

          [ **constructors** constructors ]

attributes  ::= [ decl ; ]+

decl  ::= [**series**] type id list

type  ::= primitive | class id | type[ ]

primitive ::= **real** | **int** | **bool** | **char** | **string**

id_list  ::= attribute_id [ , attribute_id ]+

constraints  ::= [ constraint ;]+

constraint  ::= creational | quantified | simple

creational  ::= attribute = **new** class_id (terms)

quantified  ::= **forall** var in enum : constraint |

**exists** var in enum : constraint

simple  ::= conditional | constraint_atom

conditional  ::= literals −− > condi body

condi body  ::= mto_literals [& mto_literals]+

mto_literals ::= literal | mto_constraint

mto_constraint  ::= **F** constraint_atom | **F** < interval > constraint_atom |

**F** < interval, interval > constraint_atom | G constraint_atom |

**G** < interval > constraint_atom |

**G** < interval, interval > constraint_atom

constraint_atom  ::= term relop term | cpred_id (terms)

relop ::= = | ! = | > | < | >= | <=

1. **Sample TCOB code representing AdEx-type neurons**

{simulation_time = 5000, start_time([adexneuron,2],[adexmodel,0])} %for 100 ms simulation time

class adexneuron

{

attributes

real C; %capacitance

% scaling Parameters

real Gleak; %Leak conductance

real Eleak; %Leak reversal potential

real Delta; % The slope factor

real Vt; %Threshold voltage

%Bifurcation Parameters

real A; % Adaptation coupling parameter

real B ; %reset adaptation value

real Tw; % Adaptation Time constant;

real Vr; %Reset voltage

series real W ; %adaptation variable

series real Vm;

series int Flag;

series real Varray;

real Iin; % Input current

real V;

real E;

real Dt;

constraints

Flag=1 --> ( C * (Vm -` Vm) /Dt=- Gleak*(`Vm -

Eleak)– `W +Iin + Gleak*Delta*pow(E,(`Vm-Vt)/Delta)) ;

Flag=1 -->(Tw*(W - `W)/Dt= A *(`Vm - Eleak )- `W) ;

Flag =1 --> Varray=Vm;

Vm > 30 --> (Vm` = Vr) ;

Vm> 30 --> (W`= W + B) ;

Vm > 30 --> Flag`=0;

Vm <= 30 --> Flag`=1 ;

Flag = 0 --> Varray = 30.0;

constructor adexneuron(Cap,GV,EE,D1,V1,A1,B1,WW,T1,Vr1,I1){

C=Cap;

Gleak=GV;Eleak=EE;Delta=D1;Vt=V1;A=A1;B=B1;

WW=W<1>;Tw=T1;Vr=Vr1;Iin=I1;

E = 2.71828;

V = -60;

Dt=0.02;

Flag<2> = 1;

Vm<1> = -60;

}

}

class adexmodel

{

attributes

adexneuron Adex;

constructor adexmodel(){

%parameters for tonic spiking

Adex = new adexneuron(200,12.0,-70.0,2.0,-50.0,

-10.0,0.0,0.0,300.0,-58.0,300.0);

dump_to_file(['Vm','W'],[Adex.Varray,Adex.W]);

}

}

1. **Compiling and executing TCOB programs**

TCOB is a constrained-object programming language, a temporal extension of the constrained-object language COB. This section contains instructions to create and run TCOB programs in SWI-prolog environment.

**SWI-prolog:**  The TCOB compiler translates a TCOB program into a CLP(R) program in the SWI-Prolog environment.In order to work with the compiler, the system must have a latest SWI-prolog installation.

You can download and install SWI-prolog from

http://www.swi-prolog.org/download/stable.

The user-manual and documentation of Swi-prolog available on

http://www.swi-prolog.org/pldoc/index.htm.

**TCOB Compiler**

o The compiler contains following Prolog files

a) tcob2swi.pl - TCOB compiler source, which translates a TCOB program into a CLP program

b) helper clpr.pl - A collection of TCOB built-in predicates.

The following section describes steps to compile the accircuit.tcob program. Here ?- is the Prolog prompt for commands.

**Step 1** Open SWI-Prolog environment and load the compiler.

$swipl

?- [tcob2swi].

**Step 2** Compile TCOB using the tcob2swi/2 command. It takes the TCOB program and driver class constructor as the input and creates the corresponding CLP(R) program.

?- tcob2swi('ac_circuit.tcob', 'samplecircuit()').

It creates a file with a .pl extension and with same name as the TCOB program.

**Step 3** Load the compiled code using standard Prolog load command.

?- [ac_circuit].

The compiler adds a main class to regulate the simulation time based on user-supplied parameters and it invokes the driver class constructor. The generated program has two arguments, the first denotes the list of attributes in the class and second denotes the list of arguments to the constructor. If you are not taking any input values, it can be represent by _ character.

?- main(_,_).

The result will be 'true' for a successful execution (all the constraints are satisfied), otherwise the result will be 'false'.

# ---------------------------------------------------------------------------------------------

To know the values of variables, we can use the following methods.

* **Using write predicates:** TCOB supports the write/1 and writeln predicates of SWI-Prolog in order to print the value of variable. The user may use these predicates in constraints, predicates and constructors of a TCOB class.

Example:

class resistor

extends component{

attributes

real R;

constraints

V = I * R;

writeln(`I);

constructor resistor(R1)

{ R = R1; }

}

* **Using constructor parameters** :A constructor can accept any number of parameters which can be used in order to get the values of variables after the execution.

Consider the following modification in the samplecircuit class in order to get the value of I, from the resistor class.

class samplecircuit {

attributes

source AC;

....

constructor samplecircuit(RI) {

R1 = new resistor(10.0);

....

RI = R1.I;

}

}

The compilation steps are as follows.

?- tcob2swi('ac_circuit.tcob', 'samplecircuit(RI)').

?- [ac_circuit].

The value of RI after execution can be obtained as follows:

?- main(_,[R1]).

R1 = [0.028415544588302557,

-0.0026938858952047884,-0.0318564641884161,-0.030040285877712104

0.0006954638679985727, 0.031661681297034526, 0.03408940847804451

0.005549458734292934]

true.

***dump_to_file(N,V)** - Dump the values of variables to a CSV file called output.csv, where N is the list of variable names and V is the list of values of variable. Each row in the CSV starts with the name of the variable.

eg: dump_to_file(['V','A], [V,A])

***plot_graph(Title,V,XLo,XHi,YLo,YHi,Width,Height,Spacing,TimeFactor)-** Plot the list of value in variable V against time. In X-axis time starts from XLo and incremented by TimeFactor unit up to XHi. YLo and YHi specifies the range of Y-axis, Width and Height are the dimensions of chart and Spacing determines the spacing of labels in X and Y axis

eg: plot_graph('Voltage',V,2, 100,-80,35,720,500,5,0.02);

1. **Modelling Excitatory and Inhibitory synapses**

Glutamatergic (excitatory) and GABAergic (inhibitory) neuro-receptors were modelled and temporal dynamics of the post-synaptic currents was represented. Excitatory synapses were modelled using AMPA and NMDA synaptic dynamics (see Equations A1_a_ –A1_f_)

$g_{AMPA(t)}= \frac{g_{AMPAMax}\times e^{\frac{-t}{18}}\times\left( 1-e^{\frac{-t}{2.2}} \right)}{0.68}$(A1_a_)

$I_{AMPA(t)}=\left( V_{m}-E_{AMPA} \right)\times g_{AMPA(t)}$ (A1_b_)

$g_{NMDA(t)}= g_{NMDAMax}\times{\frac{g}{g_{0}}\times e}^{\frac{-(t-5)}{71}}\times\frac{\left( 1-e^{\frac{-(t-5)}{13.2}} \right)}{0.60}$ (A1_c_)

$\frac{g}{g_{0}}=1-\frac{1}{1+\frac{Kmg}{{[Mg}_{0}]}}$ (A1_d_)

$Kmg=0.00107\times e^{2 \times0.73\times\frac{VF}{RT}}$(A1_e_)

$I_{NMDA(t)}=\left( V_{m}-E_{NMDA} \right)\times g_{NMDA(t)}$(A1_f_)

where${[Mg}_{0}]$ is the Magnesium concentration, *F* is the Faraday’s constant, *R* is the universal gas constant and *T* is the temperature in Kelvin.

Inhibitory synapses were modeled using GABA synaptic dynamics (see Equations A1_g –_ A1_k_)

$g_{{GABA}_{A}(t)}= \frac{g_{{GABA}_{A}Max}\times e^{\frac{-t}{25}}\times\left( 1-e^{\frac{-t}{1.0}} \right)}{0.84}$(A1_g_)

$g_{{GABA}_{B}(t)}= g_{{GABA}_{B}Max}\times({0.84 \times e}^{\frac{-t}{283}}+0.16\times e^{\frac{-t}{10226}}\times\frac{\left( 1-e^{\frac{-t}{112}} \right)^{4}}{0.31}$(A1_h_)

$I_{{GABA}_{A}(t)}=\left( V_{m}-E_{GABA} \right)\times g_{{GABA}_{A}(t)}$(A1_i_)

$I_{{GABA}_{B}(t)}=\left( V_{m}-E_{GABA} \right)\times g_{{GABA}_{B}(t)}$(A1_j_)

$I_{GABA(t)}={I_{{GABA}_{A}(t)} + I}_{{GABA}_{B}(t)}$(A1_k_)

where $E_{AMPA}, E_{NMDA}$ and $E_{GABA}$ are the reversal potential of AMPA, NMDA and GABA synapses. The synaptic currents $I_{AMPA}$,$I_{NMDA}$and $I_{GABA}$ were modelled as ohmic conductances$g_{AMPA}$,$g_{NMDA}$and $g_{GABA}$ multiplied by the driving force which was the difference between the membrane potential $V_{m}$and the reversal potential.
